# Unmasking insulinoma following commencement of somatostatin analogues in malignant neuroendocrine tumours

**DOI:** 10.1530/EO-25-0005

**Published:** 2025-07-19

**Authors:** Isuru Gamage, Emma Boehm, Gaurav Ghosh, Grace Kong, Michael Michael, HuiLi Wong, Oliver Piercey, Nirupa Sachithanandan

**Affiliations:** ^1^ Department of Internal Medicine, Peter MacCallum Cancer Centre, Melbourne, Australia; ^2^ ENETS Centre of Excellence, Peter MacCallum Cancer Centre, Melbourne, Australia; ^3^ Molecular Imaging and Therapeutic Nuclear Medicine, Cancer Imaging, Peter MacCallum Centre Melbourne, Melbourne, Australia; ^4^ Department of Clinical Pathology, Collaborative Centre for Genomic Cancer Medicine, The University of Melbourne, Melbourne, Australia; ^5^ Sir Peter MacCallum Department of Oncology, The University of Melbourne, Melbourne, Victoria, Australia; ^6^ Department of Medical Oncology, Peter MacCallum Cancer Centre, Melbourne, Australia; ^7^ Department of Medicine, The University of Melbourne, Melbourne, Australia

**Keywords:** insulinoma, neuroendocrine neoplasms, somatostatin analogue, peptide receptor radionuclide therapy, radionuclide therapy, neuroendocrine tumour, hypoglycaemia, metastatic insulinoma, metastatic neuroendocrine tumour

## Abstract

**Objective:**

Somatostatin analogues (SSA) are used in the management of patients with metastatic gastroenteropancreatic neuroendocrine tumours (GEP-NET) to control hormone secretion and tumour growth. SSA can paradoxically worsen or unmask hypoglycaemia in patients with insulinoma by inhibiting counter-regulatory hormones such as glucagon and growth hormone.

**Design and methods:**

We present two cases of SSA use in patients with initially presumed non-functioning GEP-NET unmasking insulinoma. We review the use of SSA in GEP-NET and the management of refractory hypoglycaemia in metastatic insulinoma.

**Results:**

A 62-year-old female with metastatic grade 2 GEP-NET was commenced on monthly lanreotide 10 weeks after diagnosis. She presented 1 week following the second dose with refractory hypoglycaemia and inappropriate hyperinsulinism, requiring inpatient dextrose infusion. SSA was stopped; however, she remained dextrose dependent despite the addition of diazoxide and dexamethasone. Peptide receptor radionuclide therapy (PRRT) with ^177^Lu-DOTA-Octreotate was given, resulting in resolution of hypoglycaemia after two cycles. The second case is a 57-year-old female with metastatic grade 2 GEP-NET. Four months post commencement of lanreotide, she presented with radiological disease progression and symptomatic hypoglycaemia. A 72 h fast confirmed hyperinsulinaemic hypoglycaemia. SSA was stopped. A trial of diazoxide was not tolerated, and a prednisolone trial was ineffective. The patient underwent inpatient PRRT with euglycaemia achieved shortly afterwards.

**Conclusions:**

SSA can unmask hypoglycaemia secondary to insulinoma. Detection of new-onset hypoglycaemia requires careful clinical vigilance when commencing SSA in patients with GEP-NET initially presumed to be non-functional. Hypoglycaemia from metastatic insulinoma requires multidisciplinary management incorporating nutritional, medical and oncologic therapy. PRRT can be effective in managing refractory hypoglycaemia.

**Learning points:**

- SSA use can unmask insulinoma in a NET presumed to be non-functional.
- SSA can paradoxically worsen hypoglycaemia in insulinoma due to suppression of counter-regulatory hormones.
- There are currently no biomarkers in routine clinical use to predict which patients will experience a worsening of hypoglycaemia after SSA initiation. Detection of new-onset hypoglycaemia requires clinical vigilance and education of patients to report symptoms early to enable prompt investigation and management.
- Symptomatic hypoglycaemia in metastatic insulinoma is challenging to manage and requires a multidisciplinary approach considering diet, medical therapy and urgent initiation or escalation of oncologic therapy.
- PRRT is a safe and effective strategy to achieve hormonal and oncologic control in metastatic insulinoma. Caution should be practised regarding flare of insulin release, and inpatient administration with expert endocrinology, nuclear medicine and oncology input should be considered.

## Introduction

Neuroendocrine tumours (NET) represent a rare group of malignancies with potential secretory capacity (Oronsky *et al.* 2017). Well-differentiated gastroenteropancreatic (GEP) NETs overexpress somatostatin receptors (SSTR) (de Herder & Lamberts 2002). There are five subtypes of SSTR receptors; subtypes SSTR-2 and SSTR-5 are preferentially expressed in GEP-NET (Bertherat *et al.* 2003), enabling therapeutic targeting in the form of ‘cold’ (i.e. non-radiolabelled) somatostatin analogues (SSA) octreotide and lanreotide (Rinke *et al.* 2009, Caplin *et al.* 2014), and radiolabelled SSA as peptide receptor radionuclide therapy (PRRT) (Strosberg *et al.* 2017, Singh *et al.* 2024). These are used for oncologic management and to control hormone secretion for patients with NET (Vezzosi *et al.* 2005, Vezzosi *et al.* 2008, Opalińska *et al.* 2021, Zhang *et al.* 2022, Friebe *et al.* 2024).

SSAs can paradoxically worsen hypoglycaemia in insulinoma patients due to their preferential suppression of hormones that induce hyperglycaemia (i.e. glucagon and growth hormone), which resolves post cessation of SSA therapy (Stehouwer *et al.* 1989, Abell *et al.* 2015). This has led to recommendations for slow titration and close monitoring following commencement of SSA in known insulinoma (Bertherat *et al.* 2003, Hofland *et al.* 2024). Hypoglycaemia may also occur in non-insulinoma NETs following SSA commencement via a similar mechanism (Schmid & Brueggen 2012). While rare, the conversion of an initially non-functioning NET subsequently to a functional insulinoma may occur (Wynick *et al.* 1988, de Mestier *et al.* 2015, Wu *et al.* 2022, Buddhavarapu *et al.* 2023). There is a paucity of the literature on SSA unmasking hypoglycaemia in a NET initially presumed to be non-functional. We present two patients with NET in whom SSA unmasked hypoglycaemia due to insulinoma, which was refractory to medical management but improved following treatment with ^177^Lu-DOTA-Octreotate (LuTate) PRRT.

## Case series

### Case 1

#### Case presentation

The first case is a 62-year-old female with a known history of metastatic pancreatic NET. At diagnosis, she presented with abdominal pain and weight loss, with subsequent imaging and biopsy confirming well-differentiated grade 2 pancreatic NET, Ki-67 of 10%. 10 weeks after diagnosis, she was commenced on lanreotide, 90 mg administered subcutaneously monthly.

One week after the second dose of lanreotide, the patient described new, escalating neuroglycopenic symptoms suggestive of hypoglycaemia, culminating in her presentation to her local hospital with unconscious collapse. Hypoglycaemia (blood glucose 2.6 mmol/L) was confirmed on initial assessment and managed with intravenous dextrose. Hypoglycaemia recurred within 15 min of dextrose being paused, with subsequent testing confirming inappropriate insulin secretion (Table 1). She remained dependent on intravenous dextrose to maintain euglycaemia and was transferred to our centre for further management.

**Table 1:** Fasting laboratory investigations. For case 1, laboratory tests were taken during the first admission. Hypoglycaemia developed after intravenous dextrose was paused for 15 min and was confirmed on venous glucose measurement. Additional tests were taken at the same time as the confirmatory venous glucose blood test. For case 2, laboratory tests were taken during an inpatient 72 h fast. Hypoglycaemia developed after 4 h of fasting and was confirmed on venous glucose measurement. Additional tests were taken at the same time as the confirmatory venous glucose blood test.

Test	Reference	Case 1	Case 2
Cortisol (nmol/L)	200–650	386	475
IGF-1 (nmol/L)	6–31	22.5	14
Anti-insulin antibody (U/mL)	<0.4	<0.4	<0.4
Pro-insulin (mU/L)	<13.3	>100	>100
Insulin (mU/L)	3–15	84.5	142
C peptide (nmol/L)	0.2–0.9	3.07	1.54
Ketones (mmol/L)	<0.5	0	0.2
Nadir glucose (mmol/L)		1.7	<1.0
HbA1c (%)	<6.5	Unknown	4.7
Chromogranin A (μg/L)	0–102	983	148
Gastrin (pmol/L)	6–55	816	49
Glucagon (μg/L)	25–250	Unknown	113

#### Management

**Figure 1:**
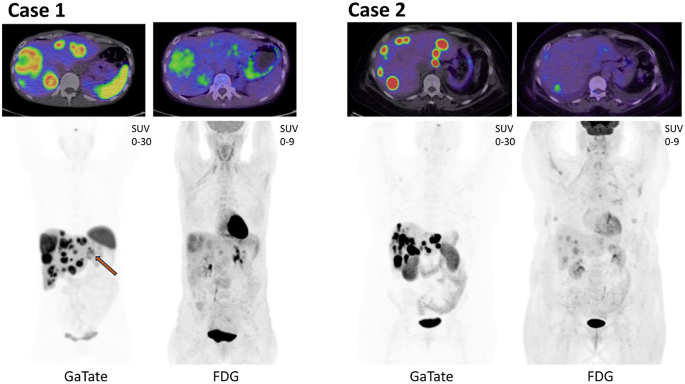
Molecular imaging phenotype of insulinoma cases. Case 1 and case 2 both demonstrate a similar imaging phenotype, with the GaTate PET/CT demonstrating ≥ Krenning 3 (3 = higher than liver, 4 = higher than spleen) SSTR expression in hepatic metastatic disease. Some hepatic lesions also have mild-to-moderate FDG avidity. Case 1: primary pancreatic lesion denoted on GaTate maximum intensity projection (MIP) by orange arrow, with the remaining disease located in the liver. The moderate FDG uptake associated with the pancreatic primary is obscured by renal activity on the MIP. Axial panels demonstrate liver disease with Krenning 3–4 SSTR expression with mild-to-moderate FDG avidity. Case 2: post distal pancreatectomy and splenectomy. No local pancreatic recurrence, multifocal hepatic metastatic disease. Axial panels demonstrate hepatic disease with Krenning 4 SSTR expression and a lesion with mild-to-moderate FDG avidity. Bilateral pleural effusions are seen on low-dose CT.

Despite the addition of diazoxide and corticosteroids and cessation of SSA, hypoglycaemia remained refractory and the patient continued to require intravenous dextrose infusion to achieve euglycaemia. A ^68^Ga-DOTA-Octreotate (GaTate) positron emission tomography/computed tomography (PET/CT) scan demonstrated a pancreatic body lesion and progressive liver metastases with high SSTR expression (Fig. 1). An ^18^F-FDG (fluoro deoxy glucose) PET/CT revealed mild-to-moderate metabolic activity within hepatic lesions and no discordant FDG-avid, SSTR-negative disease (Fig. 1). Given the severity of refractory disease and imaging phenotype, a multidisciplinary decision was made to pursue treatment with inpatient LuTate PRRT.

#### Outcome and follow-up

**Figure 2:**
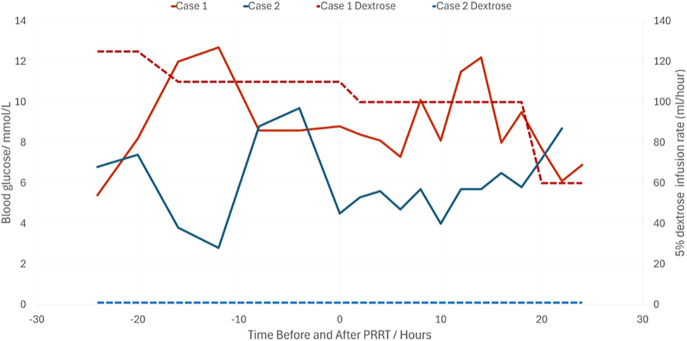
Interstitial glucose readings taken via glucometer for both cases in 24 h before and following cycle 1 of PRRT. Note stability of glucose readings for both cases following treatment, indicating no flare of insulin release.

An activity of 8.1 Gbq was administered for the first cycle of PRRT. This was tolerated well, with glycaemia and rate of dextrose administration remaining stable within 24 h of administration, suggesting no immediate flare of insulin release with therapy (Fig. 2). In the 72 h following PRRT, the IV dextrose infusion was able to be reduced and ceased without hypoglycaemia. The patient was discharged on diazoxide and oral corticosteroid, which was complicated by persistent low-grade nausea, altered taste, anorexia and weight loss. Frequent self-monitoring of blood glucose demonstrated improvement in glycaemia, with no further hypoglycaemic episodes following the first cycle of PRRT. Diazoxide and corticosteroid were weaned, leading to resolution of these symptoms without change in glycaemia. Following the second cycle, the patient achieved further sustained improvement in glycaemia. An interim response assessment demonstrated a partial molecular imaging response to therapy after the two cycles. She is planned to complete four cycles of PRRT.

### Case 2

#### Case presentation

A 62-year-old female presented with recurrent symptomatic hypoglycaemia following commencement of SSA for metastatic pancreatic NET. She was initially diagnosed with localised disease and managed with distal pancreatectomy. Histology confirmed well-differentiated grade 2 NET with a Ki-67 of 10%. Metastatic disease in the liver was discovered on surveillance CT and GaTate imaging at 3 months post-operatively. She was commenced on lanreotide 90 mg subcutaneously every 4 weeks.

At her 4-months review, the patient reported persistent new symptoms suggestive of hypoglycaemia, characterised by diaphoresis, palpitations and presyncopal symptoms, which correlated with capillary hypoglycaemia (BGL (blood glucose level) <3 mmol/L) measured with glucometer readings. Self-management with frequent carbohydrate-rich snacking led to rapid weight gain. Hyperinsulinaemic hypoglycaemia was confirmed on a 72 h fast, with nadir hypoglycaemia <1 mmol/L developing within 4 h of fasting and associated with corresponding elevated C-peptide and insulin (Table 1).

SSA was withheld and then ceased. The patient was commenced on diazoxide for symptom control and titrated to a final dose of 325 mg daily split over four doses; however, this was complicated by significant fluid retention resulting in pulmonary oedema and pleural effusions, necessitating inpatient admission for diuresis. Diazoxide was thus gradually weaned and ceased, and the patient was transitioned to 10 mg prednisolone. Despite this, euglycaemia was not achieved and the patient required intermittent administration of intravenous dextrose boluses.

#### Management

FDG and GaTate PET/CT restaging confirmed further disease progression with high SSTR expression and no discordant FDG-avid, SSTR-negative disease (Fig. 1). Given persistent symptomatic hypoglycaemia, disease progression and suitable imaging phenotype, the patient underwent urgent inpatient LuTate PRRT.

#### Outcome and follow-up

The first cycle of PRRT was well tolerated without flare in hypoglycaemia (Fig. 2). Hypoglycaemia improved rapidly, enabling discharge on prednisolone, which was ceased following her second cycle of PRRT.

## Discussion

Insulinomas are rare, with estimates of prevalence fewer than 5 per million people (Okabayashi *et al.* 2013). Diagnosis commonly occurs following presentation with symptomatic hyperinsulinaemic hypoglycaemia (Peltola *et al.* 2018). It is exceedingly rare for insulinoma to be diagnosed after an established diagnosis of clinically non-functional NET (Peltola *et al.* 2018). The ‘conversion’ of a non-functioning tumour to an insulinoma has been sparsely described in the literature (Clover *et al.* 2019, Wu *et al.* 2022, Buddhavarapu *et al.* 2023), with significant heterogeneity among tumour phenotype, time to ‘conversion’ and prior treatment. Given the paucity of cases, there is no clear consensus regarding the mechanism of ‘conversion’ to a functioning tumour. One suggested mechanism is the epigenetic modification of genetically susceptible non-functional tumours by cytotoxic therapy (Wynick *et al.* 1988, Clover *et al.* 2019). While it is possible SSA may play a similar role in genetically susceptible NETs, the short temporal course suggests the inhibition of counter-regulatory hormones such as glucagon, catecholamines and growth hormone as the most likely cause in our cases (Stehouwer *et al.* 1989, Abell *et al.* 2015). The two cases presented highlight the importance of remaining vigilant for features of hyperinsulinaemic hypoglycaemia following commencement of SSA for NET presumed to be non-functional, given the potential to ‘unmask’ previously silent tumours.

SSAs are used in patients with metastatic NET or those with localised disease who are not candidates for surgery due to their ability to inhibit hormonal secretion and slow tumour growth (Vezzosi *et al.* 2005, Vezzosi *et al.* 2008, Gomes-Porras *et al.* 2020). Both effects are due to the relative ubiquity of SSTR on well-differentiated NET cells (Gomes-Porras *et al.* 2020). There are five subtypes of SSTR (1–5), with subtypes SSTR-2 and SSTR-5 commonly expressed in GEP-NET (Bertherat *et al.* 2003). Lanreotide and octreotide are SSAs used in the management of insulinoma due to their high affinity for SSTR-2 receptors, which can be overexpressed by insulinoma cells (Vezzosi *et al.* 2005). Previous small case series have estimated efficacy in achieving euglycaemia of approximately 50% (Vezzosi *et al.* 2005, Gomes-Porras *et al.* 2020). It has been hypothesised that SSTR subtype expression plays an important role in inhibiting insulin secretion. Tumours not expressing an abundance of SSTR-2 over SSTR-5 subtypes may have reduced response to these agents and may, in fact, experience preferential suppression of glucagon over insulin, leading to hypoglycaemia (Bertherat *et al.* 2003). This has been demonstrated mechanistically by *in vivo* models (Karashima & Schally 1988, Schmid & Brueggen 2012). Given GaTate has high binding affinity to SSTR-2, expression pattern could provide further insight into estimating risk of hypoglycaemia (Maecke *et al.* 2005). However, both of our cases demonstrate high SSTR-2 expression on GaTate, and SSTR-5 was not assessed. Pasireotide is a novel multi-receptor SSA with higher affinity to SSTR-5 compared with conventional SSAs, and has been used successfully in refractory insulinomas, presenting a potential management option in tumours under-expressing SSTR-2 receptors (Tirosh *et al.* 2016, Hendren *et al.* 2018, Siddiqui *et al.* 2020, Sileo *et al.* 2020, Bolanowski *et al.* 2022). Another proposed mechanism for variability in glycaemic response to SSA use in metastatic insulinoma is the observation that SSTR receptors have the ability to associate with other molecular complexes to alter intrinsic receptor function, thereby functioning to inhibit counter-regulatory hormones (Rohrer *et al.* 1998, Cakir *et al.* 2010). Hypoglycaemia can emerge when normal liver tissue is replaced by neoplastic tissue and more commonly develops in the presence of advanced disease. Euglycaemia is maintained by increased counter-regulatory hormones and the ability of unaffected hepatic tissue to compensate through gluconeogenesis reserve (Hoff & Vassilopoulou-Sellin 1998). When an SSA is added to a susceptible GEP-NET and causes suppression of counter-regulatory hormones, euglycaemic capacity of the remaining hepatic tissue may be overwhelmed, revealing hypoglycaemia. Importantly, there is no method available currently to predict which patients will develop hypoglycaemia with SSA therapies before commencing treatment. Detection of new-onset hypoglycaemia post SSA commencement therefore requires clinical vigilance. Patients should be educated about this risk and encouraged to report neuroglycopenic/adrenergic symptoms to enable early identification and management. Trial of a short-acting SSA before use of long-acting SSA is strongly recommended in patients with known insulinoma to mitigate this risk.

The management of hypoglycaemia in insulinoma is complex, requiring an individualised, multimodal approach as observed among the two cases presented. Dietary adjustment limiting periods of prolonged fasting, with regular, frequent consumption of snacks containing slow-release carbohydrates, is integral to the initial management of insulinoma but often leads to significant weight gain (Li & Chen 2020). Dietary modification should be implemented in conjunction with medical management addressing hormone control and oncologic therapy to control tumour growth. Diazoxide, which inhibits beta cell insulin release and enhances hepatic glycogenolysis, is used to control hyperinsulinism. Small case series have estimated that diazoxide achieves glycaemic control in approximately 50% of patients with insulinoma; however, notably, these studies only included small proportions of patients with metastatic disease (Gill *et al.* 1997, Niitsu *et al.* 2019). Efficacy is likely to be lower in this cohort, mirrored by the two cases presented. Common adverse events of diazoxide include fluid retention and hirsutism, seen in approximately 50% of patients, which often lead to cessation of therapy (Shin *et al.* 2010). This was observed in case 2. Glucocorticoids can improve hypoglycaemia by increasing insulin resistance and reducing glucose uptake; however, this is associated with a significant adverse effect profile, reducing its viability in long-term management.

Oncologic therapy including chemotherapy, mammalian target of rapamycin (mTOR) inhibitors and PRRT is essential to achieve both hormonal and disease control in patients with disease not appropriate for locoregional therapies. There are no clear data to guide the choice or sequence of therapy in metastatic insulinoma. Chemotherapy options include platinum and capecitabine/temozolomide (CAPTEM) based regimens, which can achieve disease control (Frizziero *et al.* 2019, de Mestier *et al.* 2020); however, data regarding the efficacy of chemotherapy in achieving hormonal control are limited (Capdevila *et al.* 2022, Hofland *et al.* 2023). Everolimus is an mTOR inhibitor that can be used in metastatic insulinoma due to its antiproliferative activity and desirable side effect of hyperglycaemia mediated through impaired peripheral glucose uptake, impaired beta cell insulin secretion and increased hepatic gluconeogenesis (Hofland *et al.* 2023). There is a paucity of data regarding efficacy on hypoglycaemia control when taking everolimus, with one small case series reporting a 90% rate of hypoglycaemia control (Bernard *et al.* 2013). Other adverse effects which may preclude long-term use include myelosuppression and renal toxicity (Yao *et al.* 2011).

PRRT delivers targeted radiation to NETs with high SSTR expression via radiolabelled somatostatin analogues. PRRT has demonstrated improvements in progression-free survival among patients with grade 1–2 metastatic midgut NET (Strosberg *et al.* 2017) and is now FDA approved for this indication. More recently, the NETTER-2 trial has reported efficacy in grade 2–3 GEP-NET with Ki-67 of 10–55% (Singh *et al.* 2024). A recent retrospective study of 32 patients showed that PRRT resulted in improvement of symptomatic hypoglycaemia and reduction in antihypoglycaemic agents in approximately 80% of patients with metastatic insulinoma (Friebe *et al.* 2024). Further small retrospective case series have reflected similar results (Magalhães *et al.* 2019, Veltroni *et al.* 2020). Our two cases both achieved symptomatic control and cessation of all glucose-supporting medications following treatment with PRRT, reinforcing its emerging favourable profile in the management of metastatic insulinoma. Limitations of PRRT use include accessibility, cost and rare adverse events including myelosuppression and haematological malignancies (Dureja *et al.* 2023).

## Conclusions

We present two cases of metastatic insulinoma with hypoglycaemia unmasked following SSA therapy initiation. Detection of new-onset hypoglycaemia post SSA commencement requires clinical vigilance and patients should be encouraged to report symptoms early to enable prompt identification and management. Both cases presented challenges in managing symptomatic hypoglycaemia in metastatic insulinoma. PRRT with LuTate was effective to ameliorate hypoglycaemia in both cases, enabling safe patient discharge from hospital. The use of a multimodal approach to manage refractory hypoglycaemia in patients with metastatic NET remains imperative, involving a combination of diet, medical therapy and urgent initiation of oncological therapy.

## Declaration of interest

The authors declare that there is no conflict of interest that could be perceived as prejudicing the impartiality of the work reported.

## Funding

This work did not receive any specific grant from any funding agency in the public, commercial or not-for-profit sector.

## Author contribution statement

IG, EB and NS wrote the first draft of the manuscript and all authors edited, reviewed and approved the final version of the manuscript.

## References

[bib1] Abell SK, Teng J, Dowling A, et al. 2015 Prolonged life-threatening hypoglycaemia following dose escalation of octreotide LAR in a patient with malignant polysecreting pancreatic neuroendocrine tumour. Endocrinol Diabetes Metab Case Rep 2015 14–0097. (10.1530/edm-14-0097) 25755880 PMC4313612

[bib2] Bernard V, Lombard-Bohas C, Taquet M-C, et al. 2013 Efficacy of everolimus in patients with metastatic insulinoma and refractory hypoglycemia. Eur J Endocrinol 168 665–674. (10.1530/eje-12-1101) 23392213

[bib3] Bertherat J, Tenenbaum F, Perlemoine K, et al. 2003 Somatostatin receptors 2 and 5 are the major somatostatin receptors in insulinomas: an in vivo and in vitro study. J Clin Endocrinol Metab 88 5353–5360. (10.1210/jc.2002-021895) 14602773

[bib4] Bolanowski M, Kałużny M, Witek P, et al. 2022 Pasireotide – a novel somatostatin receptor ligand after 20 years of use. Rev Endocr Metab Disord 23 601–620. (10.1007/s11154-022-09710-3) 35067849 PMC9156514

[bib5] Buddhavarapu VS, Dhillon G, Grewal HS, et al. 2023 Transformation of pancreatic nonfunctioning neuroendocrine tumor into metastatic insulinoma: a rare case report. Clin Case Rep 11 e8152. (10.1002/ccr3.8152) 37942181 PMC10627923

[bib6] Cakir M, Dworakowska D & Grossman A 2010 Somatostatin receptor biology in neuroendocrine and pituitary tumours: part 2 – clinical implications. J Cell Mol Med 14 2585–2591. (10.1111/j.1582-4934.2010.01125_1.x) 20629988 PMC4373478

[bib7] Capdevila J, Grande E, García-Carbonero R, et al. 2022 Position statement on the diagnosis, treatment, and response evaluation to systemic therapies of advanced neuroendocrine tumors, with a special focus on radioligand therapy. Oncologist 27 1–12. (10.1093/oncolo/oyab041) 35380724 PMC8982404

[bib8] Caplin ME, Pavel M, Ćwikła JB, et al. 2014 Lanreotide in metastatic enteropancreatic neuroendocrine tumors. N Engl J Med 371 224–233. (10.1056/nejmoa1316158) 25014687

[bib9] Clover T, Abdelkader A & Guru Murthy GS 2019 Transformation of a non-secretory neuroendocrine tumor to insulinoma after treatment with sunitinib: a case report and review of the literature. J Oncol Pharm Pract 25 1516–1519. (10.1177/1078155218791309) 30089432

[bib10] de Herder WW & Lamberts SW 2002 Somatostatin and somatostatin analogues: diagnostic and therapeutic uses. Curr Opin Oncol 14 53–57. (10.1097/00001622-200201000-00010) 11790981

[bib11] de Mestier L, Hentic O, Cros J, et al. 2015 Metachronous hormonal syndromes in patients with pancreatic neuroendocrine tumors: a case-series study. Ann Intern Med 162 682–689. (10.7326/m14-2132) 25984844

[bib12] de Mestier L, Walter T, Evrard C, et al. 2020 Temozolomide alone or combined with capecitabine for the treatment of advanced pancreatic neuroendocrine tumor. Neuroendocrinology 110 83–91. (10.1159/000500862) 31071715 PMC6979423

[bib13] Dureja S, McDonnell M, Van Genechten D, et al. 2023 Global challenges in access to diagnostics and treatment for neuroendocrine tumor (NET) patients. J Neuroendocrinol 35 e13310. (10.1111/jne.13310) 37351944

[bib14] Friebe L, Freitag MT, Braun M, et al. 2024 Peptide receptor radionuclide therapy is effective for clinical control of symptomatic metastatic insulinoma: a long-term retrospective analysis. J Nucl Med 65 228–235. (10.2967/jnumed.123.265894) 38164592 PMC13671384

[bib15] Frizziero M, Spada F, Lamarca A, et al. 2019 Carboplatin in combination with oral or intravenous etoposide for extra-pulmonary, poorly-differentiated neuroendocrine carcinomas. Neuroendocrinology 109 100–112. (10.1159/000497336) 30703770

[bib16] Gill GV, Rauf O & MacFarlane IA 1997 Diazoxide treatment for insulinoma: a national UK survey. Postgrad Med J 73 640–641. (10.1136/pgmj.73.864.640) 9497974 PMC2431498

[bib17] Gomes-Porras M, Cárdenas-Salas J & Álvarez-Escolá C 2020 Somatostatin analogs in clinical practice: a review. Int J Mol Sci 21 1682. (10.3390/ijms21051682) 32121432 PMC7084228

[bib18] Hendren NS, Panach K, Brown TJ, et al. 2018 Pasireotide for the treatment of refractory hypoglycaemia from malignant insulinoma. Clin Endocrinol 88 341–343. (10.1111/cen.13503) 29055143

[bib19] Hoff AO & Vassilopoulou-Sellin R 1998 The role of glucagon administration in the diagnosis and treatment of patients with tumor hypoglycemia. Cancer 82 1585–1592. (10.1002/(sici)1097-0142(19980415)82:8<1585::aid-cncr22>3.3.co;2-z) 9554538

[bib20] Hofland J, Refardt JC, Feelders RA, et al. 2023 Approach to the patient: insulinoma. J Clin Endocrinol Metab 109 1109–1118. (10.1210/clinem/dgad641) PMC10940262 37925662

[bib21] Hofland J, Refardt JC, Feelders RA, et al. 2024 Approach to the patient: insulinoma. J Clin Endocrinol Metab 109 1109–1118. (10.1210/clinem/dgad641) 37925662 PMC10940262

[bib22] Karashima T & Schally AV 1988 Superactive somatostatin analog decreases plasma glucose and glucagon levels in diabetic rats. Peptides 9 561–565. (10.1016/0196-9781(88)90164-7) 2901737

[bib23] Li R & Chen W 2020 Ground-breaking application of raw corn starch-based diet in patients with insulinoma-related hypoglycemia: a before-after prospective study. Curr Dev Nut 4(Supp. 2) 541. (10.1093/cdn/nzaa046_041)

[bib24] Maecke HR, Hofmann M & Haberkorn U 2005 ^68^Ga-labeled peptides in tumor imaging. J Nucl Med 46 172S–178S. 15653666

[bib25] Magalhães D, Sampaio IL, Ferreira G, et al. 2019 Peptide receptor radionuclide therapy with 177Lu-DOTA-TATE as a promising treatment of malignant insulinoma: a series of case reports and literature review. J Endocrinol Investig 42 249–260. (10.1007/s40618-018-0911-3) 29949120

[bib26] Niitsu Y, Minami I, Izumiyama H, et al. 2019 Clinical outcomes of 20 Japanese patients with insulinoma treated with diazoxide. Endocr J 66 149–155. (10.1507/endocrj.ej18-0353) 30504655

[bib27] Okabayashi T, Shima Y, Sumiyoshi T, et al. 2013 Diagnosis and management of insulinoma. World J Gastroenterol 19 829–837. (10.3748/wjg.v19.i6.829) 23430217 PMC3574879

[bib28] Opalińska M, Sowa-Staszczak A, Maraih IA, et al. 2021 Peptide receptor radionuclide therapy as a tool for the treatment of severe hypoglycemia in patients with primary inoperable insulinoma. Bio Algorithm Med Syst 17 221–226. (10.1515/bams-2021-0138)

[bib29] Oronsky B, Ma PC, Morgensztern D, et al. 2017 Nothing but NET: a review of neuroendocrine tumors and carcinomas. Neoplasia 19 991–1002. (10.1016/j.neo.2017.09.002) 29091800 PMC5678742

[bib30] Peltola E, Hannula P, Huhtala H, et al. 2018 Characteristics and outcomes of 79 patients with an insulinoma: a nationwide retrospective study in Finland. Int J Endocrinol 2018 2059481. (10.1155/2018/2059481) 30425741 PMC6218736

[bib31] Rinke A, Müller HH, Schade-Brittinger C, et al. 2009 Placebo-controlled, double-blind, prospective, randomized study on the effect of octreotide LAR in the control of tumor growth in patients with metastatic neuroendocrine midgut tumors: a report from the PROMID study group. J Clin Oncol 27 4656–4663. (10.1200/JCO.2009.22.8510) 19704057

[bib32] Rohrer SP, Birzin ET, Mosley RT, et al. 1998 Rapid identification of subtype-selective agonists of the somatostatin receptor through combinatorial chemistry. Science 282 737–740. (10.1126/science.282.5389.737) 9784130

[bib33] Schmid HA & Brueggen J 2012 Effects of somatostatin analogs on glucose homeostasis in rats. J Endocrinol 212 49–60. (10.1530/joe-11-0224) 21987782

[bib34] Shin JJ, Gorden P & Libutti SK 2010 Insulinoma: pathophysiology, localization and management. Future Oncol 6 229–237. (10.2217/fon.09.165) 20146582 PMC3498768

[bib35] Siddiqui M, Vora A, Ali S, et al. 2020 Pasireotide: a novel treatment for tumor-induced hypoglycemia due to insulinoma and non-islet cell tumor hypoglycemia. J Endocr Soc 5 bvaa171. (10.1210/jendso/bvaa171) 33294765 PMC7692539

[bib36] Sileo F, Cangiano B, Cacciatore C, et al. 2020 Off-label pasireotide treatment in one insulinoma patient with an atypical presentation and intolerant to diazoxide. Endocrine 70 435–438. (10.1007/s12020-020-02406-1) 32621049

[bib37] Singh S, Halperin D, Myrehaug S, et al. 2024 [^177^Lu]Lu-DOTA-TATE plus long-acting octreotide versus highdose long-acting octreotide for the treatment of newly diagnosed, advanced grade 2–3, well-differentiated, gastroenteropancreatic neuroendocrine tumours (NETTER-2): an open-label, randomised, phase 3 study. Lancet 403 2807–2817. (10.1016/s0140-6736(24)00701-3) 38851203

[bib38] Stehouwer CD, Lems WF, Fischer HR, et al. 1989 Aggravation of hypoglycemia in insulinoma patients by the long-acting somatostatin analogue octreotide (Sandostatin). Acta Endocrinol 121 34–40. (10.1530/acta.0.1210034) 2545062

[bib39] Strosberg J, El-Haddad G, Wolin E, et al. 2017 Phase 3 trial of ^177^Lu-Dotatate for midgut neuroendocrine tumors. N Engl J Med 376 125–135. (10.1056/nejmoa1607427) 28076709 PMC5895095

[bib40] Tirosh A, Stemmer SM, Solomonov E, et al. 2016 Pasireotide for malignant insulinoma. Hormones 15 271–276. (10.14310/horm.2002.1639) 26732164

[bib41] Veltroni A, Cosaro E, Spada F, et al. 2020 Clinico–pathological features, treatments and survival of malignant insulinomas: a multicenter study. Eur J Endocrinol 182 439–446. (10.1530/eje-19-0989) 32061159

[bib42] Vezzosi D, Bennet A, Rochaix P, et al. 2005 Octreotide in insulinoma patients: efficacy on hypoglycemia, relationships with Octreoscan scintigraphy and immunostaining with anti-sst2A and anti-sst5 antibodies. Eur J Endocrinol 152 757–767. (10.1530/eje.1.01901) 15879362

[bib43] Vezzosi D, Bennet A, Courbon F, et al. 2008 Short- and long-term somatostatin analogue treatment in patients with hypoglycaemia related to endogenous hyperinsulinism. Clin Endocrinol 68 904–911. (10.1111/j.1365-2265.2007.03136.x) 18031316

[bib44] Wu LW, Qasim Hussaini SM, Lee JW, et al. 2022 Transformation of metastatic nonfunctioning pancreatic neuroendocrine tumor into insulinoma – two case reports. Ann Pancreat Cancer 5 6. (10.21037/apc-22-1)

[bib45] Wynick D, Williams SJ & Bloom SR 1988 Symptomatic secondary hormone syndromes in patients with established malignant pancreatic endocrine tumors. N Engl J Med 319 605–607. (10.1056/nejm198809083191003) 2842676

[bib46] Yao JC, Shah MH, Ito T, et al. 2011 Everolimus for advanced pancreatic neuroendocrine tumors. N Engl J Med 364 514–523. (10.1056/nejmoa1009290) 21306238 PMC4208619

[bib47] Zhang D, Lu L, Zhu HJ, et al. 2022 Somatostatin treatment for ectopic ACTH syndrome due to pancreatic neuroendocrine tumors: review of the literature. Int J Endocrinol 2022 6283706. (10.1155/2022/6283706) 35265125 PMC8901294

